# Comparison of efficacy in adjuvant chemotherapy regimens in patients with radically resected gastric cancer: a propensity-matched analysis

**DOI:** 10.18632/oncotarget.11783

**Published:** 2016-09-01

**Authors:** RenCui Quan, JiaXing Huang, HongTao Chen, YiFeng Liao, WeiZe Lv, Nan Chen, JianJun Liu, HongYu Zhang, DaZhi Xu

**Affiliations:** ^1^ Department of Medical Oncology, The Fifth Affiliated Hospital of Sun Yat-Sen University, Zhu Hai, Guangdong Province, People's Republic of China; ^2^ Department of Laboratory, The Fifth Affiliated Hospital of Sun Yat-Sen University, Zhu Hai, Guangdong Province, People's Republic of China; ^3^ Department of Gastric and Pancreatic Surgery, Sun Yat-sen University Cancer Center, Guangzhou, Guangdong Province, China; ^4^ State Key Laboratory of Oncology in South China, Collaborative Innovation Center for Cancer Medicine, Guangzhou, Guangdong Province, China

**Keywords:** adjuvant chemotherapy, radically resected, gastric cancer

## Abstract

**Background:**

We conducted the retrospective study to compare the efficacy of monotherapies versus two-drug regimens as postoperative chemotherapy for patients with radically resected gastric cancer.

**Result:**

At a median follow-up of 5.3 years, no significant difference in terms of OS was observed between two groups, neither before nor after matching. After matching, median DFS was statistically significant between group A and B (median, 67.5 vs 101.0 months, respectively; hazard ratio [HR], 0.65; 95% CI, 0.45 to 0.95; *P*=0.027), which meant doublets prolonged DFS. In subgroup analysis, the patients of stage III receiving doublet achieved better OS than those receiving monotherapy. People who received doublet and were less than 65 years old, or male patients, or in T4 stage, or in N2 stage, or receiving subtotal gastrectomy had better DFS than those with monotherapy.

**Method:**

A data set including 501 patients (monotherapy, n=107; doublet, n=394) was matched between the two groups (n=107 patients per group) using the propensity-matched study. The primary and secondary endpoint was overall survival(OS) and disease-free survival(DFS), respectively. Survival data was compared using the Kaplan-Meier method and Cox proportion hazards models for univariate and multivariate analyses.

**Conclusion:**

The dual regimens seemed not to add overall survival benefits to patients receiving curative gastrectomy, compared with single-agent fluoropyrimidine as postoperative chemotherapy. However, dual regimens showed better disease-free survival.

## INTRODUCTION

Gastric cancer not only is the fifth most common malignancy around the word, after cancers of the lung, breast, colorectum and prostate, but also the third leading cause of cancer-related deaths [1]. Numerous randomized trials and meta-analyses have authenticated the survival advantage of adjuvant chemotherapy and chemo-radiotherapy in patients with curatively resected gastric cancer [2–8]. However, there are no consistent standard postoperative chemotherapy regimens. Triplet regimens (epirubicin, cisplatin, and 5-fluororacil) or adjuvant chemo-radiotherapy are well accepted in European and American countries where D0 and D1 resections are preferred choices, based on evidence from the MAGIC and INT-0116 trials [3, 6]. In oriental countries where D2 resection is routine, especially in Japan and China, clinicians prefer to administrate S-1 single agent or oxaliplatin combined with capecitabine based on the results from the ACTS-GC and CLASSIC studies [2, 4]. The ACTS-GC study reported that postoperative adjuvant chemotherapy with S-1 alone reduced the risk of death by 33%. The overall survival rate at 5 years was 71.7% in the S-1 group and 61.1% in the surgery-only group [4]. Meanwhile, the CLASSIC trial confirmed that adjuvant chemotherapy with capecitabine and oxaliplatin (XELOX) after D2 surgery for patients from Asia with stage II or III gastric cancer resulted in significantly higher disease-free survival (DFS) [2]. The 3 year DFS was 74% in the XELOX group and 59% in the surgery only group. All the patients in CLASSIC and ACTS-GC trials had undergone D2 node resection. However, we can not compare with these two trials because their endpoints were 5-year survival rate and 3-year disease-free survival, respectively. Another randomized phase III trial (ARTIST trial) suggested that the addition of XRT (capecitabine plus radiotherapy) to XP (capecitabine plus cisplatin) chemotherapy did not significantly reduce recurrence after curative resection and D2 lymph node dissection in gastric cancer [9]. Furthermore, a randomized trial to investigate the efficacy and toxicity of FOLFOX4 and LV5Fu2 regimens in patients with advanced gastric adenocarcinoma after curative gastrectomy demonstrated that the FOLFOX4 group were better than the 5-FU/LV group in 3-year recurrence free and the 3-year overall survival rate [10]. The study shown a statistically significant overall survival for FOLFOX4 group over control group, with a median 3-yr RFS and 3-yr OS of 30.0 and 36.0 moths for FOLFOX4 group, versus 16.0 and 28.0 months in the control group, respectively. However, another randomized trial (ITACA-S study) which was designed to evaluate the efficacy of a sequential treatment of FORFORI followed by docetaxel plus cisplatin in comparison to 5-FU/LV demonstrated that no statistically significant difference was between multi-regimen and monotherapy in both disease-free and overall survival [11]. Moreover, another trial showed that S-1 plus paclitaxel was not superior to paclitaxel alone in second-line chemotherapy in terms of PFS and OS [12]. These approaches produce inconsistent survival results, and the optimal adjuvant therapy for patients of gastric cancer with radical gastrectomy has not been established.

In our retrospective study, in an effort to identify the standard postoperative adjuvant chemotherapy for gastric cancer, we compared single agents with dual regimens in terms of overall survival and disease-free survival in patients with radically resected gastric cancer.

## RESULTS

### Matched patient characteristics

The median age was 59 years (ranging 19 to 85 years), with 73 male patients (68.2%), 52 patients (48.6%) with poorly differentiated adenocarcinoma, and 94 patients (87.9%) received subtotal gastrectomy in matched patients of group A. Meanwhile, in group B, the median age was 57 years (ranging from 19 to 86 years), with 73 male patients (68.2%), 56 patients (52.3%) with poorly differentiated adenocarcinoma, and 88 patients (82.2%) received subtotal gastrectomy. All the patients were treated with D2 lymphadenectomy. Before matching, there were significant differences in variables of lymph nodes stage and TNM stage. However, they were well balanced after matching (Table 1). The regimens were clearly listed (Table 2). In group A, 52 patients (48.6%) had been administrated capecitabine, and in group B, 74 patients (69.1%) had received oxaliplatin with a fluoropyrimidine (FOLFOX:32.7%, XELOX:36.4%); The median number of treatment cycles delivered was 5 (ranging 1-28) and 5 (ranging1-10) in group A and B, respectively.

**Table 1:** The Characteristics of the patients

	Before matching			After matching		
	Monotherapy	Doublet	*P*-value^a^	Monotherapy	Doublet	*P*-value^a^
Total	107	394		107	107	
Age(years)						
<65	76	309	.108	76	76	1.000
≥65	31	85		31	31	
Gender						
Male	73	252	.412	73	75	.767
Female	34	142		34	32	
T stage						
T1	8	16	.116	8	11	.542
T2	18	41		18	17	
T3	25	114		25	32	
T4	56	222		56	47	
N stage						
N0(0)	48	80	.000^b^	48	36	.377
N1(1-2)	21	78		21	24	
N2(3-6)	15	93		15	21	
N3(≥7)	23	143		23	26	
TNM Stage						
I	22	19	.000^b^	22	14	.251
II	37	128		37	46	
III	48	247		48	47	
Type of resection						
Total gastrectomy	9	59	.134	9	17	.094
Subtotal gastrectomy	97	335		97	90	
Histology						
Well differentiated	32	97	.580	32	29	.867
Poorly differentiated	52	194		52	56	
Signet-ring cell	17	82		17	18	
Others	6	21		6	4	
Location of tumor						
Cardia	34	104	.306	34	37	.340
Antrum	49	174		49	10	
Corpus	15	57		15	44	
Others	9	59		9	16	

a Chi-square test;

b statistically significant;

T stage: Depth of primary tumor invasion; N stage: Regional lymph nodes classification; Others in Histology: Mucinous adenocarcinoma and adenosquamous carcinoma; Others in Location tumor: Mixture of cardia and corpus or mixture of corpus and antrum or the whole stomach.

**Table 2:** Adjuvant chemotherapy regimens administered in the two groups of patients

Group A	N. patients		Group B	N. patients	
Capectabine	52	(48.6%)	FOLFOX	35	(32.7%)
S-1	8	(7.5%)	XELOX	39	(36.4%)
Carmofur	5	(4.7%)	Taxans+fluoropynidine	21	(19.6%)
FT207	18	(16.8%)	Platium-based	10	(9.3%)
UFT	16	(15%)	Etoposide+fluoropynidine	2	(1.9%)
5-FU	8	(7.5%)			

FT207(Tegafur); UFT(Tegafur-uracil); 5-FU (5-Fluorouracil); FOLFOX (Oxaliplatin, 5-fluorouracil, leucovorin); XELOX (Xeloda,Oxaliplatin), Taxanes (paclitaxel or docetaxel).

### Overall survival and disease-free survival

**Figure 1:**
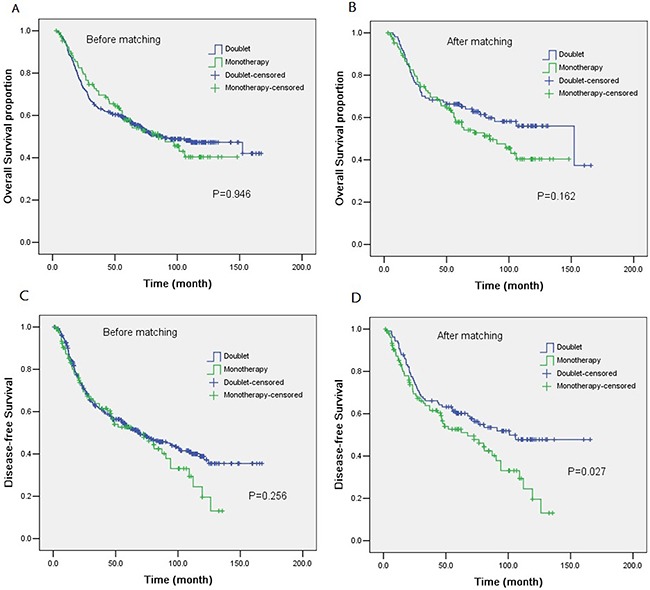
Kaplan-Meier curves of overall survival (OS) and disease-free survival (DFS) according to the chemotherapy regimens The P value for the difference between the two curves was determined by log-rank test. Notes: **A.** OS for patients before matching. **B.** OS for patients after matching. **C.** DFS for patients before matching. **D.** DFS for patients after matching.

By the last follow-up time on December 30^th^ 2014, the median follow-up time was 63.3 months and 65 months in group A and group B, respectively. 99 patients (92.5%) in group A and 102 patients (95.3%) in group B had finished the follow-up time. In monotherapy group, 58 patients (54.2%) were confirmed to have recurrent disease, and 52 patients (48.6%) died. Whereas, in the doublet group, 50 patients (46.7%) were confirmed to relapse, and 44 patients (41.1%) died. Before matching, among the 501 patients, the median OS were 84.4 months and 86.3 months (HR 0.99, 95%CI: 0.73-1.35) in group A and B, respectively(*P*=0.946). Median DFS was 67.5 months with group A and 69.0 months with group B (HR, 0.85; 95% CI, 0.63 to 1.13; *P*=0.256). However, after matching, median DFS was significantly better in group B (101.0 months) than that in group A (67.5 months; HR, 0.65; 95%CI, 0.45 to 0.95; *P*=0.027). There was no significant difference between the two groups in terms of OS (Figure 1).

Univariable analysis identified six significant prognostic variables associated with overall survival (Table 3), Age(*P*=0.001), T stage(*P*=0.005), N stage(*P*=0.000), TNM stage(*P*=0.000), Type of resection(*P*=0.001) and Location of tumor(*P*=0.001). In particular, Multivariate Cox proportional hazards regression analysis identified that, regimens of therapy, age, location of tumor, type of resection and N stage (all *P*<0.05) were associated with OS (Table 4). However, only Age, TNM stage and N stage were related with DFS (Table 4).

**Table 3:** Univariable analysis of disease-free survival and overall survival

Factor	*P* Value
Disease-Free Survival(DFS)	
Regimens of chemotherapy	.027
Age	.006
Gender	.447
T stage	.005
N stage	.000
TNM stage	.000
Type of resection	.052
Histology	.188
Location of tumor	.058
Overall Survival(OS)	
Regimens of chemotherapy	.162
Age	.001
Gender	.972
T stage	.005
N stage	.000
TNM stage	.000
Type of resection	.001
Histology	.327
Location of tumor	.001

Regimens of chemotherapy : Monotherapy or two-drug regimens; T stage: Depth of primary tumor invasion; N stage: Regional lymph nodes classification.

**Table 4:** Multivariate analysis of Disease-free survival and Overall Survival

Variables	Hazard Ratio	95%CI		*P*^a^
		LL	UL	
**Disease-free survival**				
Age				
<65	Reference			
≥65	1.90	1.27	2.83	.002^b^
TNM stage				
IB	Reference			
II	1.82	0.76	4.37	.180
III	3.15	1.28	7.72	.012^b^
N stage				
N0	Reference			
N1	0.90	0.48	1.71	.750
N2	1.52	0.81	2.88	.194
N3	2.36	1.28	4.36	.006^b^
**Overall Survival**				
Regimens of therapy				
monotherapy	Reference			
two-drug regimens	0.55	0.35	0.85	.008^b^
Age				
<65	Reference			
≥65	2.14	1.39	3.28	.001^b^
Location of tumor				
cardia	Reference			
corpus	0.45	0.20	1.02	.056
antrum	0.54	0.34	0.86	.009^b^
Type of resection				
subtotal gastrectomy	Reference			
total gastrectomy	2.39	1.31	4.39	.005^b^
N stage				
N0	Reference			
N1	0.86	0.43	1.72	.669
N2	2.52	1.38	4.59	.003^b^
N3	4.45	2.61	7.59	.000^b^

Abbreviations: N stage: Regional lymph nodes classification; LL =lower limit; UL=upper limit.

a Cox regression analysis.

b Statistically significance.

### Subgroup analysis

**Figure 2:**
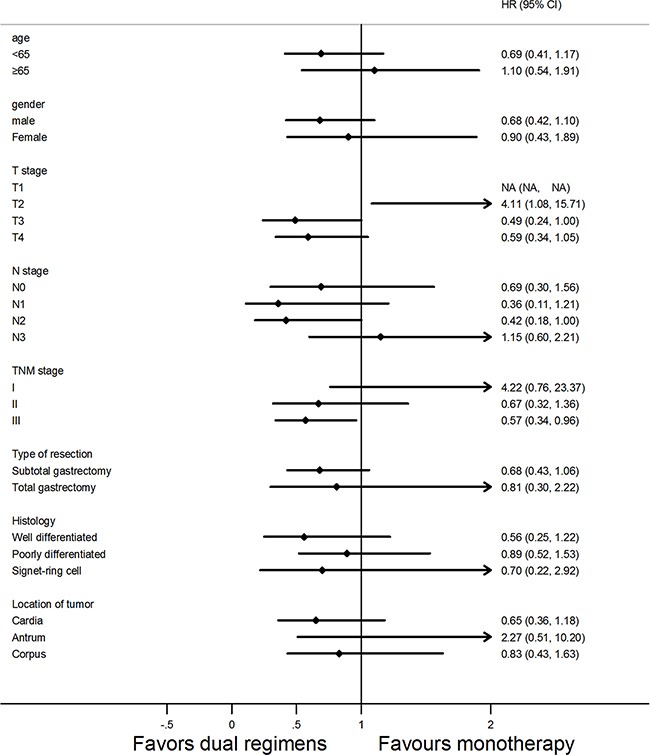
Forest plot of overall survival (OS) according to the regimens of adjuvant chemotherapy in subgroup analysis Abbreviations: HR, hazard ratio, HR <1 implies a lower risk of death for patients; 95% CI, 95% confidence intervals.

**Figure 3:**
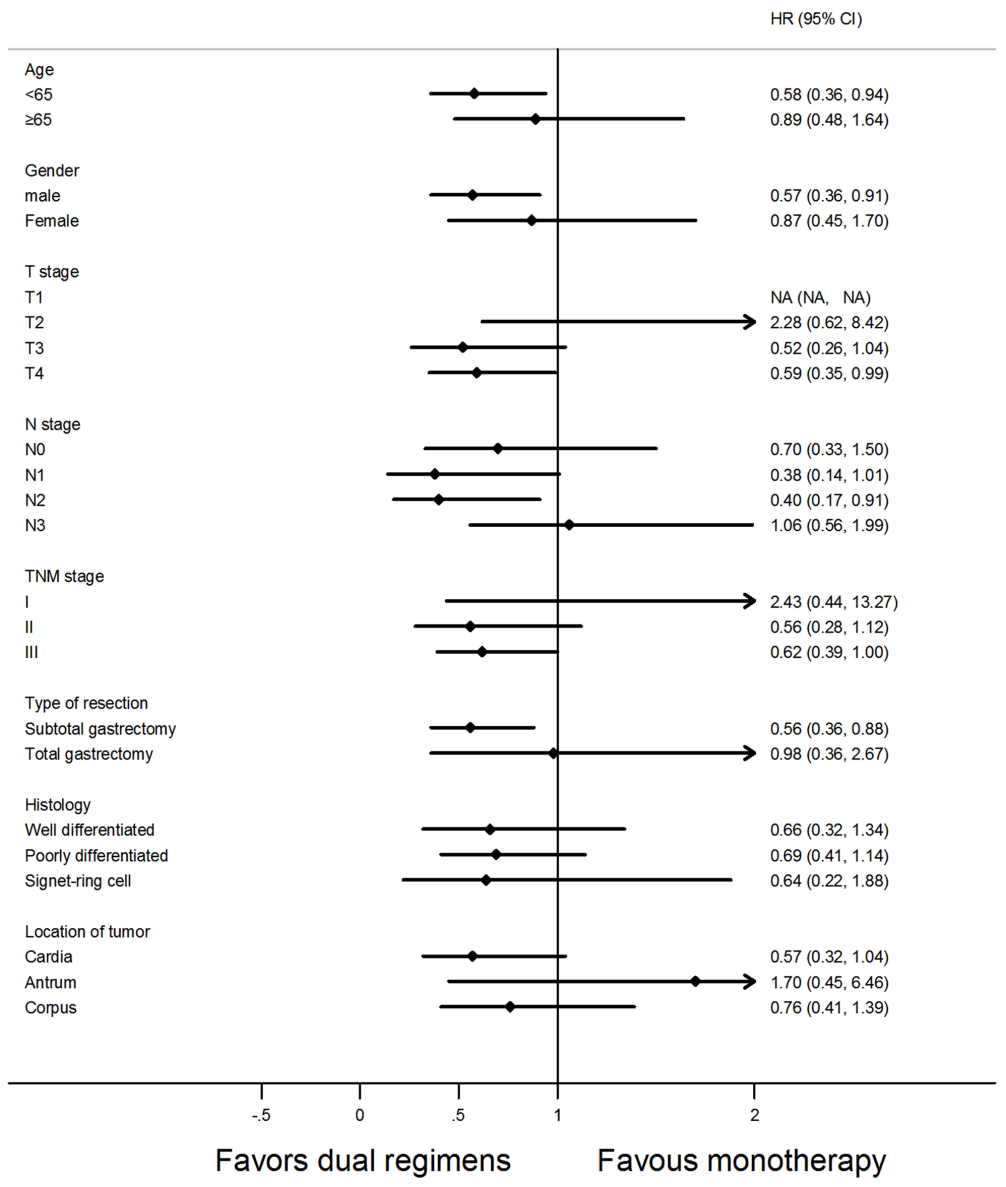
Forest plot of disease-free survival (DFS) according to the regimens of adjuvant chemotherapy in subgroup analysis Abbreviations: HR, hazard ratio, HR <1 implies a lower risk of relapse for patients; 95% CI, 95% confidence intervals.

Overall and disease-free survival were analyzed according to age, gender, disease stage, type of resection, histology type and location of tumor. None of the variables but patients in stage III receiving doublets obtained marked benefits in terms of OS (Figure 2). Importantly, when examining predictors of disease-free survival, in group B, the patients with less than 65 years old, or in T4 stage, or in N2 stage, or male patients, or receiving subtotal gastrectomy had better DFS than those in group A (Figure 3).

## DISCUSSION

It is well accepted that adjuvant chemotherapy after curative resection contributes survival benefits to gastric cancer treatment [5, 14, 15]. Whether intensive multi-chemotherapy would achieve satisfactory results when being compared to monotherapy remains attractive.

The characteristics of patients (Table 1) showed that N stage and TNM stage were statistically different between the two arms before matching. It was likely that the imbalance of TNM stage influenced the results of survival analysis [2, 4]. Therefore, we performed a propensity analysis. Moreover, after matching, the multivariate analysis indicated that N stage not only had a positive correlation with OS but also with DFS, which confirmed the propensity-score analysis was necessary. After matching, all the variables were well balanced between the two arms. However, at a median follow-up of 5.3 years, no significant difference was found between the two groups in terms of OS, neither before nor after matching. Subgroup analysis showed that the patients of stage III received doublets achieved better overall survival than those in monotherapy (Figure 2). Maybe the patients with advanced stage would benefit from dual regimens, and for those with tumors in early stage, monotherapy turns to be a better choice. Markedly, after matching, median DFS was statistically significantly different between arm A and arm B (median, 67.5 vs 101.0 months, respectively; HR, 0.95; *P*=0.027), which was consistent with the results from previous trial [10, 11]. Consistently, a meta-analysis by Iacovelli et al, reported a significant reduction of risk of death and relapse by 13% and 23% in combined chemotherapy, respectively, when compared to monotherapy [16]. However, a significant heterogeneity was found, which was a significant limit of the above study. In particular, the chemotherapy regimens in the meta-analysis consisting of several types of combination or sequential therapies may attributed to the encouraging benefits. Interestingly, people who received two-drugs with an age less than 65 years, or male patients, or in T4 stage, or in N2 stage, or receiving subtotal gastrectomy had a better DFS than those in monotherapy (Figure 3). Looking at the results of the forest plots, an important stratified factor able to discriminate between patients who benefit from monotherapy versus intensive chemotherapy is the location of the primary tumor. It seems that patients with tumor located in the antrum fail to benefit from doublets in terms of DFS and OS. According to the study presented by Scartozzi M et al, different biological subtypes of tumors lead to varied sensitivity to chemotherapy treatments. They proposed a new classification for gastric cancer(GC), based on Lauren' s histology and on anatomic tumor, identifying three subtypes of disease: type 1(proximal non diffuse GC), type 2 (diffuse GC), and type 3 (distal non diffuse GC) [17]. Proximal gastric cancer is associated with the chronic Helicobacter pylori infection and they seem to reduce the risk of death in some case-control cohort studies [18, 19]. In particular, another study confirmed that gene polymorphisms may affect the chemotherapy sensitivity and metastatic process in gastric cancer [20]. These studies suggested that subtypes of gastric cancer may be important predictors for patients with gastric cancer who could benefit from chemotherapy. In clinical practice, oncologists are more likely to prescribe single agent chemotherapy for older patients or patients at earlier stage according to their physical condition [21, 22]. However, our result demonstrated that intensive two-drug chemotherapy regimen is better than mild chemotherapy in disease-free survival after curative resection with gastric cancer. From the Kaplan-Meier curves of overall survival, the two curves clearly separated after median overall survival, but with no significant difference. Probably more participates and longer follow-up can help separated the whole curves completely. Our result is in line with another observational study which may point out the direction [23]. It is hypothesized that the lack of a significant difference in overall survival between patients treated with three or two drugs in first line, may be associated with the influence of second-line or thereafter treatments. Moreover, the benefit of salvage treatments in patients with gastric cancer after progression to first-line chemotherapy was well recognized [24–26]. Nevertheless, our study failed to collect the adverse events between two arms due to the loss of records. Yet, several trials showed that the adverse events in both monotherapy and two-drug regimens were manageable [10, 11, 27].

The propensity-score analysis made our study convincing. Otherwise, the major limit of our study is the small sample size after matching and selection bias of the samples. Single-center and retrospective design are another two limitations of our study.

In conclusion, this retrospective study was complementary to phase III prospective trials which compared the efficacy and safety between different regimens. Our study demonstrated that dual regimens are a better choice for patients with high risk factors as stage III, and N2 stage.

## PATIENTS AND METHODS

### Patients

To evaluate whether a two-drug regimen could be comparable to a single-agent approach as postoperative adjuvant chemotherapy in patients after curative gastrectomy, a retrospective analysis was conducted in Sun Yat-Sen cancer center. Between January 2000 and December 2010, a total of 501 gastric carcinoma patients who received radical surgery and followed by adjuvant chemotherapy were enrolled. The inclusive criteria were as follows: Patients aged 18 years or order with histologically confirmed stomach carcinoma; curative resection with D2 lymph node dissections; no evidence of distant metastases; TNM stage of IB-IIIC (according to the seventh edition of American Joint Committee on Cancer (AJCC) TNM Staging Classification for Carcinoma of the Stomach); no previous malignancies; and no chemotherapy or radiotherapy prior to surgery.

The inclusive patients were divided into two groups, A and B, based on the postoperative adjuvant chemotherapy regimens used (group A=monotherapy; group B=two drugs).

### Statistical methods

The Chi-square test was used to compare the characteristics between the two groups. Survival analysis was performed using the Kaplan-Meier method and compared using the log-rank test. All the statistical tests were two-sided. Meanwhile, Cox proportional hazards regression models were used to assess the effects of potential predictive variables on the DFS and OS with 95%CIs. Age, gender, T stage, N stage, TNM stage, type of resection, histology, and location of tumor were entered into a Cox regression model with forward stepwise selection of covariates. The threshold of entering and removing limits was 0.05 and 0.10, respectively.

Disease-free survival (DFS) was calculated as the period from the date of surgery to the date of recurrence or the last follow-up date. Overall survival (OS) was defined as the period from the date of surgery to the date of death of any cause or until last follow-up. All statistical analyses were performed with the SPSS, version 13.0 (Chicago, IL, USA).

### Propensity score analysis

Propensity score matching is a tool for causal inference and adjust a treatment effect for measure confounders in non-randomized studies using SPSS. The software generates estimation of propensity score using logistic regression and specifying nearest-neighbor matching with all covariates. Detailed balance statistics and relevant graphs are produced by the program“PS Matching”in SPSS [13]. In the present study, Age, Gender, T stage, N stage, TNM stage, Type of resection, Histology and Location of tumor were entered into a non-parsimonious multivariable logistic regression model. By this means, 107 of 107 patients who underwent monotherapy were matched with 107 of 394 patients who were treated with doublet with similar propensity scores (Table 1). Patient demographics and tumor characteristics for the matched groups were listed (Table 1).
